# AvrRxo1 Is a Bifunctional Type III Secreted Effector and Toxin-Antitoxin System Component with Homologs in Diverse Environmental Contexts

**DOI:** 10.1371/journal.pone.0158856

**Published:** 2016-07-08

**Authors:** Lindsay R. Triplett, Teja Shidore, John Long, Jiamin Miao, Shuchi Wu, Qian Han, Changhe Zhou, Hiromichi Ishihara, Jianyong Li, Bingyu Zhao, Jan E. Leach

**Affiliations:** 1 Department of Plant Pathology and Ecology, The Connecticut Agricultural Experiment Station, New Haven, CT, United States of America; 2 Department of Bioagricultural Sciences and Pest Management and Program in Plant Molecular Biology, Colorado State University, Fort Collins, CO, United States of America; 3 Department of Horticulture, Virginia Polytechnic Institute and State University, Blacksburg, VA, United States of America; 4 Department of Biochemistry, Virginia Polytechnic Institute and State University, Blacksburg, VA, United States of America; 5 Laboratory of Tropical Veterinary Medicine and Vector Biology, College of Agriculture, Hainan University, Haikou, 570228, Hainan, China; University of Manchester, UNITED KINGDOM

## Abstract

Toxin-antitoxin (TA) systems are ubiquitous bacterial systems that may function in genome maintenance and metabolic stress management, but are also thought to play a role in virulence by helping pathogens survive stress. We previously demonstrated that the *Xanthomonas oryzae* pv. *oryzicola* protein AvrRxo1 is a type III-secreted virulence factor that has structural similarities to the zeta family of TA toxins, and is toxic to plants and bacteria in the absence of its predicted chaperone Arc1. In this work, we confirm that AvrRxo1 and its binding partner Arc1 function as a TA system when expressed in *Escherichia coli*. Sequences of *avrRxo1* homologs were culled from published and newly generated phytopathogen genomes, revealing that *avrRxo1*:*arc1* modules are rare or frequently inactivated in some species and highly conserved in others. Cloning and functional analysis of *avrRxo1* from *Acidovorax avenae*, *A*. *citrulli*, *Burkholderia andropogonis*, *Xanthomonas translucens*, and *Xanthomonas euvesicatoria* showed that some AvrRxo1 homologs share the bacteriostatic and Rxo1-mediated cell death triggering activities of AvrRxo1 from *X*. *oryzae*. Additional distant putative homologs of *avrRxo1* and *arc1* were identified in genomic or metagenomic sequence of environmental bacteria with no known pathogenic role. One of these distant homologs was cloned from the filamentous soil bacterium *Cystobacter fuscus*. *avrRxo1* from *C*. *fuscus* caused watersoaking and triggered Rxo1-dependent cell collapse in *Nicotiana benthamiana*, but no growth suppression in *E*. *coli* was observed. This work confirms that a type III effector can function as a TA system toxin, and illustrates the potential of microbiome data to reveal new environmental origins or reservoirs of pathogen virulence factors.

## Introduction

Toxin-antitoxin (TA) systems are bacterial stress-management modules consisting of an antibacterial protein toxin and a neutralizing protein or RNA antitoxin. TA systems were first recognized as a mechanism for plasmid maintenance, and were proposed to use a post-segregational killing mechanism: when plasmid encoding a TA system is lost, the antitoxin would be degraded and the remaining toxin kills the plasmid-free cell [1]. Analyses of bacterial genomes later revealed that TA systems are ancient and ubiquitous among free-living bacteria. They are diverse, with over 20 families of TA systems in five major classes, and highly abundant, with up to 97 systems in a single genome [2,3]. Bacterial genomes have also revealed that the vast majority of TA systems reside on bacterial chromosomes [2], demonstrating that plasmid maintenance is not the only role for these modules. TA systems are now understood to play an important role in managing bacterial responses to environmental stress [4]; stress conditions may regulate TA system expression directly or indirectly [5,6]. Some TA modules have been associated with an increased prevalence of persister cells, or cells that survive antibiotic treatments [7]. Finally, it has been found that TA systems may play a critical role in host-pathogen interactions. Deletion of the *sehAB* TA system from *Salmonella typhimurium* [8] or one of three TA systems from uropathogenic *Escherichia coli* [9] partially or fully compromises virulence in mouse models, hypothetically due to the loss of stress resistance advantages conferred by the TA systems. The release of a TA toxin after lysis of intracellular *Rickettsia* bacteria was connected to early apoptosis in the host cells [10]. However, direct secretion of a TA system toxin into a eukaryotic host has never been demonstrated.

AvrRxo1-ORF1 is a secreted effector of *Xanthomonas oryzae* pv. *oryzicola* (*Xoc*), the causal agent of bacterial leaf streak of rice. It triggers a type III secretion-dependent hypersensitive resistance response (HR) in maize or transgenic rice plants expressing the resistance protein Rxo1 [11,12]. AvrRxo1-ORF1 is encoded upstream of a second open reading frame encoding AvrRxo1-ORF2, which was hypothesized to be a secretion chaperone. Recently, we reported the solved structure of AvrRxo1-ORF1 in complex with AvrRxo1-ORF2, and found that AvrRxo1-ORF1 has a structure and catalytic sites conserved among T4 polynucleotide kinase domain proteins [13]. Inactivation of kinase catalytic sites disrupted the virulence effect of AvrRxo1-ORF1 during *Xanthomonas* infection of rice, and inhibited the watersoaking phenotype caused by transient expression of AvrRxo1-ORF1 in plant leaves. AvrRxo1-ORF2 has a structure atypical of type III secretion chaperones and binds to AvrRxo1-ORF1 in a way predicted to occlude the ATP-binding site [13].

The AvrRxo1-ORF1 structure showed a strong similarity to the *Streptococcus pyogenes* plasmid pSM19035-encoded zeta (ζ) toxin [13], which forms a TA pair with its cognate antitoxin epsilon (ε). Members of the zeta/epsilon family of TA system were originally found on multiple resistance plasmids in human pathogenic *Enterococcus* and *Streptococcus* species [14,15], but homologs have since been shown to reside in diverse bacterial phyla and on chromosomes [3,16]. Unlike the majority of characterized TA toxins, which inhibit proteins involved in polynucleotide replication or translation, zeta toxins are small molecule kinases that inhibit bacterial cell wall synthesis through inactivating phosphorylation of UDP-N-acetylglucosamine [17]. A zeta toxin locus is strongly associated with virulence in *Streptococcus pneumoniae* isolates, although the basis for this connection is unknown (reviewed in [18]). Consistent with a potential role as a TA toxin, AvrRxo1-ORF1 suppressed bacterial growth when expressed in the absence of AvrRxo1-ORF2 in *E*. *coli* [13]. However, this study did not demonstrate suppression of AvrRxo1-ORF1 activity by -ORF2 expression from a separate complementary vector, and thus did not conclusively identify AvrRxo1-ORF1 as a TA system toxin. Furthermore, AvrRxo1-ORF1 activity has been reported in other phytopathogenic species [19], and it is not known whether homologous genes in these organisms may also form functional toxins.

In this study, we rename AvrRxo1-ORF1 and -ORF2 as AvrRxo1 and Arc1 (*A*vrRxo1 *r*equired *c*haperone), respectively. Using comparative structural modeling and a TA system validation experiment, we demonstrate that AvrRxo1 and Arc1 comprise a functional TA system similar to members of the zeta-epsilon family. Bacterial genomic sequence was used to analyze and clone homologs of *X*. *oryzae* AvrRxo1 from other *Xanthomonas*, *Burkholderia*, and *Acidovorax* species. Although homologs have divergent sequences, genomic contexts, and bacterial growth suppression activities, all but one were able to trigger a rapid Rxo1-dependent cell death in *Nicotiana benthamiana*. We also examine *avrRxo1*:*arc1 –*like modules revealed through recent genome and metagenome studies of environmental microorganisms, and demonstrate that an *avrRxo1* homolog from the soil myxobacterium *Cystobacter fuscus* is able to trigger AvrRxo1-like watersoaking and Rxo1-dependent defense responses when expressed in tobacco. This study supports recent findings that effectors may have environmental roles outside the host-pathogen context, and suggests that the increasing availability of whole-genome microbiome data is likely to yield new insights regarding effector origins.

## Results

### AvrRxo1 has a structure and predicted catalytic residues conserved with Zeta toxins, and functions with Arc1 as a toxin-antitoxin system in *E*. *coli*

**Fig 1:**
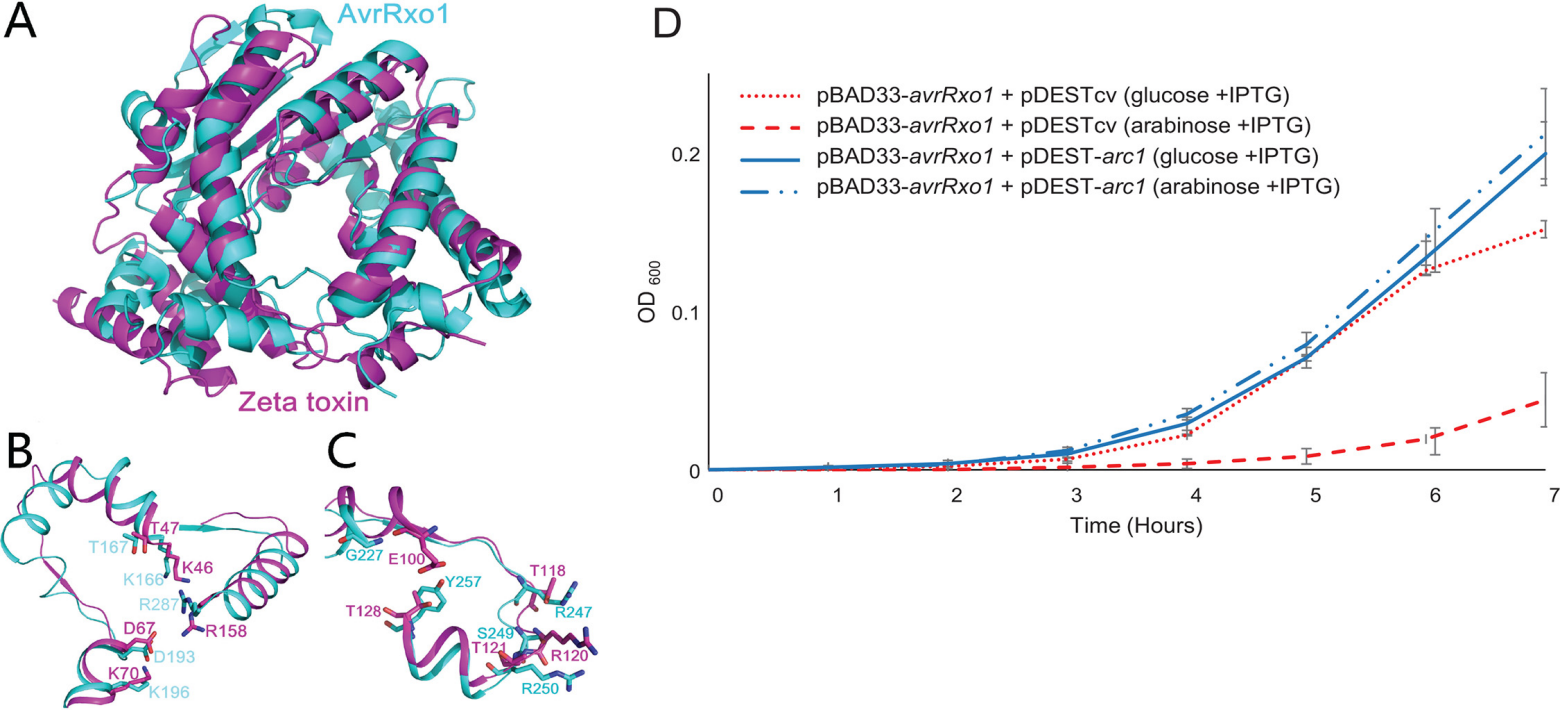
AvrRxo1:Arc1 form a novel toxin-antitoxin system in the zeta:epsilon superfamily. **A)** Alignment of the structures of AvrRxo1 (teal) and zeta toxin (magenta) demonstrate a conserved architecture. **B)** Alignment of ATP-binding domain shows complete conservation of functional residues, while **C)** only one of five residues predicted to coordinate the substrate are identical. **D)** Growth curves of *E*. *coli* BL21(DE3) co-transformed with pBAD33-*avrRxo1* and either pDEST-*arc1* or a pDEST control vector (pDESTcv) under conditions of *avrRxo1* induction (arabinose) or repression (glucose). Data points and error bars represent the means and standard deviations of nine cultures. Growth experiments were performed three times on separate days with similar results.

Our previous study demonstrated that AvrRxo1 has structural similarity to zeta toxin of *Streptococcus pyogenes* [13]. Here, we superimposed the two structures to determine the extent of similarity between AvrRxo1 and zeta toxin (Fig 1A). Five key zeta toxin kinase catalytic residues are conserved or similar in AvrRxo1: AvrRxo1 K166 (K46 in zeta toxin), T167 (T47), D193 (D67), K196 (K70), and R287 (R158) (Fig 1B). These features suggest AvrRxo1 may have a catalytic activity similar to that of zeta toxin, which phosphorylates the peptidoglycan precursor UDP-N-acetylglucosamine (UDP-GlcNAc) to form inactive UDP-GlcNAc -3P [17]. However, the UDP-GlcNAc binding residues of zeta toxin [17] (E100, T118, R120, T121 and T128) and the corresponding putative substrate binding residues in AvrRxo1 (G227, R247, S249, R250, and Y257) share little identity (Fig 1C). These observations are consistent with our previous findings that AvrRxo1 does not phosphorylate the UDP-GlcNAc substrate [13], and suggest that AvrRxo1 could have a different target or molecular activity.

We previously demonstrated that a clone carrying the *avrRxo1* gene suppresses the growth of *E*. *coli*, while a clone carrying a longer fragment encompassing *avrRxo1*:*arc1* is not growth suppressive. However, because this assay compared two separate *avrRxo1* constructs, it was not sufficient to conclude that Arc1 expression is the factor suppressing the bacteriostatic phenotype of AvrRxo1 and confirm AvrRxo1:Arc1 as a TA system. To confirm this, we employed a standard validation assay in which AvrRxo1 and Arc1 from *Xoc* were expressed from separate plasmids, pBAD33 and pDEST527, under control of arabinose and IPTG-inducible promoters, respectively. In *E*. *coli* co-transformed with the pBAD33- *avrRxo1(Xoc)* and a control vector, growth was repressed in arabinose-amended induction media compared with in glucose-amended repressing media (Fig 1D). However, in *E*. *coli* co-transformed with pBAD33-*avrRxo1* and pDEST*-arc1*, there was no difference between the growth curves in *avrRxo1*-inducing and repressing media (Fig 1D). These results demonstrate that Arc1 acts as an antitoxin suppressing the bacteriostatic effects of AvrRxo1, establishing that AvrRxo1 and Arc1 function as a TA toxin-antitoxin system in *E*. *coli*.

### AvrRxo1:Arc1 distribution and genomic context in plant pathogenic species

TA systems are thought to function in prevention of their own loss, so we hypothesized that the AvrRxo1:Arc1 system might be broadly distributed in related species. A BLAST search using the sequence of *avrRxo1* from *Xoc* (henceforth *avrRxo1-Xoc*) as a query identified annotated homologs in the sequenced genomes of *X*. *translucens*, *X*. *euvesicatoria*, *X*. *alfalfae*, *Acidovorax avenae*, and *A*. *citrulli*. To determine the *avrRxo1* sequence from the Rxo1-triggering strain *Burkholderia andropogonis* Ba3549, we sequenced and assembled a draft genome of this strain, then resequenced the predicted open reading frame with the closest homology to *avrRxo1*. *avrRxo1* homologs from complete genomes are all encoded upstream of a small open reading frame similar in size similar to that of *arc1* in *Xoc* (Table 1), with predicted products sharing 61 to 83% amino acid identity to *arc1*. All *avrRxo1* homologs have a GC content lower than that of the respective host genomes, and have differing flanking genes (Table 1), suggesting that the *avrRxo1* homologs were obtained through horizontal transfer after species diversification.

**Table 1:** Gene characteristics of predicted *avrRxo1* homologs.

Species^a^	*avrRxo1* product	*arc1* product	Identity to AvrRxo1-*Xoc*	AvrRxo1 length	Arc1 length	%GC of operon	%GC of genome	Upstream ORF	Downstream ORF
*Xoc*	AEQ98135	AEQ98134	—	421	98	50.5	64.1	IS1114 transposase	IS1114 transposase
*Aa*	WP_013594643	WP_013594642	55	414	98	59.9	68.8	Bacteriocin biosynthesis protein	Polyketide biosynthesis protein
*Ac*	ABM33628	ABM33629	55	414	98	59.9	68.5	Bacteriocin biosynthesis protein	Polyketide biosynthesis protein^b^
*Ba*	ALF40614	WP_024906130	55	422	98	53.8	58.9	Integrase	Unknown^c^
*Xe*	CAJ26159	CAJ26160	87	450	98	50.2	64.7	Hypothetical protein	Hypothetical protein
*Xac*	AEO44435	AEO44436	85	378	98	49.7	64.9	Hypothetical protein	Hypothetical protein^b^
*Xad*	WP_057682231	WP_057682238	86	450	98	50.4	64.9	Restriction endonuclease	Hypothetical protein
*XoUS*	USX11_RS0121535^d^	WP_029217965	86	Pseudo-gene	98	47.9	64.1	Unknown^c^	Unknown^c^
*Xt*	CCP41911	CCP41910	62	418	98	53.2	68.6	Hypothetical protein	FolB

^*a*^ *Xoc*, *X*. *oryzae* pv. *oryzicola* strain BLS256; *Aa*, *A*. *avenae* strain ATCC19860; *Ac*, *A*. *citrulli* strain AAC00-1; Ba, *B*. *andropogonis* strain Ba3549; Xe, *X*. *euvesicatoria* strain 85–10; *Xac*, *X*. *alfalfae* subsp. *citrumelonis* strain F1; *Xad*, *X*. *axonopodis* pv. *dieffenbachiae* LMG12749, *XoUS*, *X*. *oryzae* US strain X11-5A; Xt, *X*. *translucens* strain DSM18974.

^b^The predicted genes flanking avrRxo1:arc1 in Ac are homologous to flanking genes in Aa, and the predicted genes flanking the module in Xac are homologous to flanking genes in Xe.

^c^ The *avrRxo1*-*arc1* modules from the *Ba* and XoUS genomes are close to the end of short contigs, and genomic context is unknown.

^d^ *avrRxo1* from XoUS is a disrupted pseudogene, while *arc1* is intact.

The intraspecies distribution of *avrRxo1* has previously been examined by amplification or gene sequencing studies in Asian strains of *Xoc* ([12], 40 positives of 40 strains tested), African strains of *Xoc* ([20], 2 of 16 tested), and global strains of *Acidovorax citrulli* ([21], present in 22 of 22 strains, but inactivated by a single site mutation in 14 strains). Since the publication of these studies, the draft sequences of 74 additional strains of *X*. *euvesicatoria*, *X*. *oryzae*, and *X*. *translucens* have been published. We also accessed unpublished genomes of 12 strains of *A*. *avenae* including 10 isolated from turf (Quan Zeng, personal communication). A search against all draft (wgs) genome sequences in the families *Burkholderiaceae*, *Enterobacteriaceae*, and *Xanthomonadaceae* revealed that *X*. *alfalfae* and some *X*. *axonopodis* pathovars also have *avrRxo1*. In the 99 whole and draft genomes available for these pathogen species or pathovars, 59 *avrRxo1* homologs were identified (S1 Table). Genome analysis was supplemented by PCR screening of additional isolates in our collection of *B*. *andropogonis*, *A*. *avenae*, *X*. *euvesicatoria*, *X*. *translucens*, and 31 strains of *A*. *citrulli* not tested in previous studies. Genome sequences and PCR analysis demonstrated that intact *avrRxo1* open reading frames are completely distributed among strains of *X*. *euvesicatoria* and strains from the Asian clade of *X*. *oryzae* pv. *oryzicola* (Table 2, S1 Table). However, in the maize and cereal pathogens *B*. *andropogonis*, *A*. *avenae*, and *X*. *translucens*, only a minority of strains have an *avrRxo1* gene. Many or all strains of *A*. *citrulli*, *X*. *alfalfae*, and other clades of *X*. *oryzae* harbor *avrRxo1*, but these are inactivated by a single-site mutation or transposon inactivation in some strains (Table 2, S1 Table). The *arc1* gene is intact downstream of all *avrRxo1* homologs, including pseudogenes. In strains of *A*. *citrulli* and *X*. *oryzae* in which *avrRxo1* is inactivated by a transposon insertion, the *avrRxo1* portion has accumulated numerous mutations upstream of the transposon, but none downstream (S1 Fig). This suggests that while *avrRxo1* is frequently inactivated in some species, that there may be selective pressure favoring conservation of the gene module, perhaps in order to preserve expression of *arc1*. The *avrRxo1*:*arc1* module shares flanking genes within each species (S1 Table), suggesting genomic context of the module is stable within species.

**Table 2:** Summary of distribution analysis of *avrRxo1* in plant pathogenic bacteria.

Species	Hosts	*# avrRxo1* genomes/total (# pseudogenes)	*# avrRxo1* PCR positive strains/total	Total proportion containing *avrRxo1*
*Acidovorax avenae* (non-turf)	maize, rice, sugarcane	5/5 (0)	5 of 10	67%
*Acidovorax citrulli*	melon	4/4 (3)	31/31	100% (i)^a^
*Burkholderia andropogonis*	maize, sorghum, sugarcane	1/2 (0)	4 of 14	23%
*Xanthomonas alfalfae*	alfalfa, citrus	3/3 (2)	NT	100% (i)
*X*. *axonopodis* pv. *allii*	onion	1/1 (1)	NT	100% (i)
*X*. *axonopodis* pv. *dieffenbachiae*	anthurium, philodendron	1/4 (0)	NT	25%
*X*. *euvesicatoria*	pepper	29/29 (0)	5 of 5	100%
*X*. *oryzae* pv. *oryzicola*, Asian	rice	9/9 (0)	8 of 8	100%
*X*. *oryzae* pv. *oryzicola*, African	rice	1/3 (1)	NT	33% (i)
*X*. *oryzae*, US clade	rice	2/2 (2)	NT	100% (i)
*X*. *translucens*	wheat, barley, forage	3 of 29 (0)	2 of 16	11%

^a^ (i) indicates that some *avrRxo1* genes are inactivated in this species.

In inoculation tests on Rxo1 (B73) and non-Rxo1 (Mo17) maize, using *Acidovorax* and *Burkholderia* strains, only *B*. *andropogonis* produced either HR or watersoaking on maize. A subset of *B*. *andropogonis* strains were also tested for *avrRxo1* presence by PCR; while all strains carrying *avrRxo1* triggered HR on B73 maize, three *B*. *andropogonis* strains in which no *avrRxo1* could be detected also caused HR in maize (S2 Table). A Southern blot using a *B*. *andropogonis avrRxo1* probe in one of these strains, B7a, also failed to detect *avrRxo1* (S2 Fig). This suggests that some strains of *B*. *andropogonis* have divergent homologs of *avrRxo1* or an alternate mechanism of triggering Rxo1. Alternately, these strains might be triggering a second resistance mechanism present in B73 but absent in Mo17. The Southern blot also revealed the presence of two copies of *avrRxo1* in strain Ba3549, something not evident from the draft genome assembly.

#### Plant pathogen homologs of AvrRxo1 share bacteriostatic activity and plant toxicity functions

**Fig 2:**
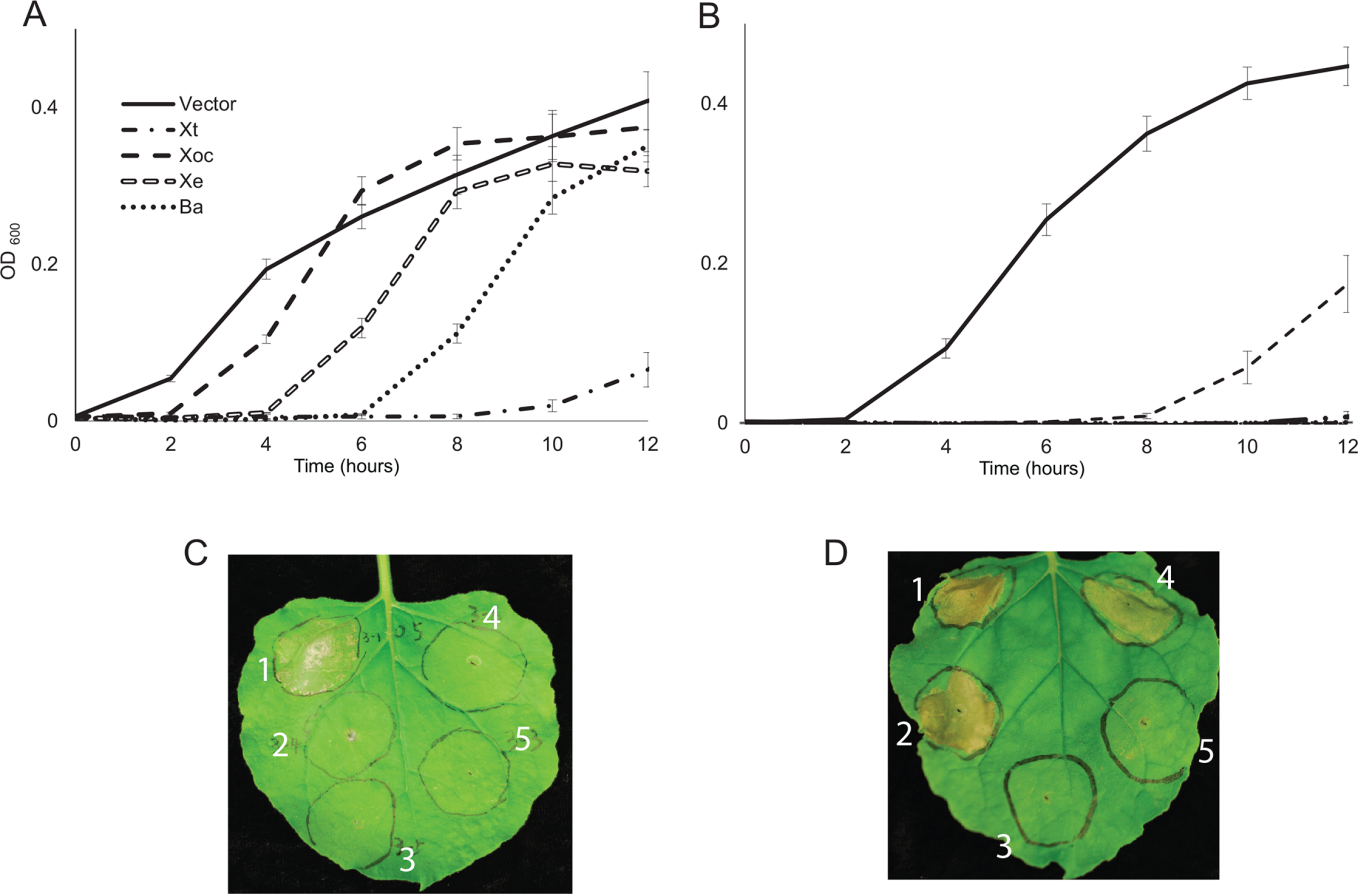
**Homologs of AvrRxo1 from multiple species suppress *E*. *coli* growth (A,B) and induce Rxo1-dependent cell death when transiently expressed in tobacco (C, D). A**) Growth of BL21(DE3) carrying pDEST-AvrRxo1-*Xoc*, -*Xe*, -*Ba*, or -*Xt*, or pDESTcv, starting at 10^7^ CFU/mL. **B**) Growth of the same strains starting at 10^6^ CFU/mL. Error bars represent the standard deviation of 16 replicate cultures. Experiments were repeated three times on different days. **C**) *N*. *benthamiana* leaves four days after infiltration with *Agrobacterium* expressing YFP fusions of AvrRxo1-*Xoc* (1), -*Ba*(2), -*Ac* (3), and *Xe* (4), or YFP alone (5). **D**) *N*. *benthamiana* leaf in which the homologs from **C** were co-expressed with a YFP fusion of Rxo1, imaged two days after agroinfiltration.

We previously reported that AvrRxo1-*Xoc* suppresses bacterial growth when expressed in *E*. *coli* cultures [13]. Here, we cloned homologs from four additional species (*X*. *translucens*, *avrRxo1-Xt*; *X*. *euvesicatoria*, *avrRxo1*-*Xe*; *B*. *andropogonis*, *avrRxo1*-*Ba*, and *A*. *citrulli*, *avrRxo1*-*Ac*) into the pDEST527 IPTG-inducible expression vector to determine whether their products had the same activities. In several attempts, no colonies lacking inactivating mutations could be obtained after transformation of the pDEST-*avrRxo1*-*Ac* clone into the expression *E*. *coli* strain BL21(DE3). The other four cloned AvrRxo1 homologs suppressed *E*. *coli* growth compared with a vector control upon IPTG-inducible expression (Fig 2A and 2B). Growth suppression phenotypes were more pronounced in cultures induced from a lower initial density (10^6^ CFU/mL, Fig 2B) than from a higher density (10^7^ CFU/mL, Fig 2A). *avrRxo1* genes from *X*. *translucens* and *B*. *andropogonis* delayed log phase longer than the genes from *X*. *euvesicatoria* or *X*. *oryzae*, suggesting that the homologs from the *Xanthomonas* clade may have lost features required for maximum growth suppressing activity.

For assessment of *in planta* activity, four of the homologs were transiently expressed as YFP fusions in *N*. *benthamiana*. Expression of the AvrRxo1-*Xoc* YFP fusion caused a clear watersoaking phenotype followed by cell collapse after four days, consistent with previous reports of phytotoxic activity [22], but expression of YFP fusions of AvrRxo1-*Ba*, -*Ac*, and–*Xe* did not (Fig 2C). When co-infiltrated with a binary construct expressing the corresponding resistance gene *Rxo1*, the AvrRxo1-*Xoc* YFP fusion elicited a strong rapid cell collapse visible after 24 h. This response was clearly differentiated from the *N*. *benthamiana* response to AvrRxo1-*Xoc* alone in its severity and rapid appearance, indicating that Rxo1-mediated cell death can be reconstituted in *N*. *benthamiana*. This phenotype was shared by the constructs expressing fusions of AvrRxo1-*Ba* and–*Xe*, but not by AvrRxo1-*Ac* (Fig 2D). Confocal images were taken to confirm that all fusions were expressed *in planta* (S3 Fig). Together, these results indicate that avirulence activity is conserved in diverse homologs of AvrRxo1, but that AvrRxo1 bacterial growth suppressing activity is not directly correlated to phytotoxicity in plant cells.

#### *avrRxo1-*like and *arc1*-like predicted genes from environmental bacteria share TA system gene organization

A protein BLAST search using the sequence of AvrRxo1*-Xoc* revealed predicted proteins sharing at least 38% positive substitutions over at least 65% of the length of AvrRxo1 in the genomes of six species of bacteria from diverse environments, including a filamentous *Myxobacterium* and two unculturable bacteria characterized through metagenome sequencing (Table 3). The genes from candidate metagenome genera *Saccharimonas* and *Parcubacteria* are predicted to encode proteins significantly longer than AvrRxo1, with the AvrRxo1 similarity focused in the central domain. Interestingly, these longer homologs are also predicted to contain C-terminal Fic/Doc domains, adenylation domains associated with the Doc family of type II toxin-antitoxin systems. The putative *avrRxo1* genes from *C*. *fuscus*, *Methylibium sp*. CF468, and *Parcubacteria* sp. SG8_24 are each followed immediately by a short open reading frame in the same direction, consistent with the organization of the *avrRxo1*:*arc1* toxin-antitoxin system. *Candidatus S*. *aalborgensis* also has an *arc1*-like short open reading frame downstream of the putative *avrRxo1* gene, although there is another open reading frame separating these. The products of these short ORFs are predicted to share >20% identity to *arc1*-*Xoc* or to the *C*. *fuscus* homolog (Table 3).

**Table 3:** Putative distant homologs of *avrRxo1* and *arc1* from environmental bacteria.

		upstream *avrRxo1-like ORF*			downstream *arc1-like ORF*		
Strain	Source	ID of predicted product^a^	Length (aa)	Identity/positive substitutions vs. AvrRxo1 (%)	ID of predicted product	Length (aa)	Identity to Arc1 (%)
*C*. *fuscus* DSM2262	Soil	EPX56639	378	30/46	EPX56640	81	33%
*Methylibium* sp. CF468	Populus root endosphere	WP_052211388	408	52/70	WP_047502103	98	55%
*Parcubacteria* sp. SG8_24	Sediment metagenome	KPJ86019	893	35/55	KPJ86020	115	None^b^
*Cand*. *S*. *aalborgensis*	Sewer sludge metagenome	AGL61921	742	36/52	AGL61919	121	21%
*Endozoicomonas numazuensis*	Marine sponge symbiont	WP_0348346	625	23/38	N/A	N/A	N/A
*Leptospira broomii*	Human disease	none			WP_040912049	96	39%
*Nocardia rhamnosiphila*	Compost	none			WP_039817270	97	44%
*Corallococcus coralloides*	Soil	none			WP_014395978	97	41%

^*a*^ Predicted products of “*avrRxo1*- like” genes align to AvrRxo1 with greater than 65% query coverage and an e-value smaller than 1e-60.

^b^The predicted product shares 42% identity with *C*. *fuscus* EPX56640 and contains the conserved double tyrosine motif.

**Fig 3:**
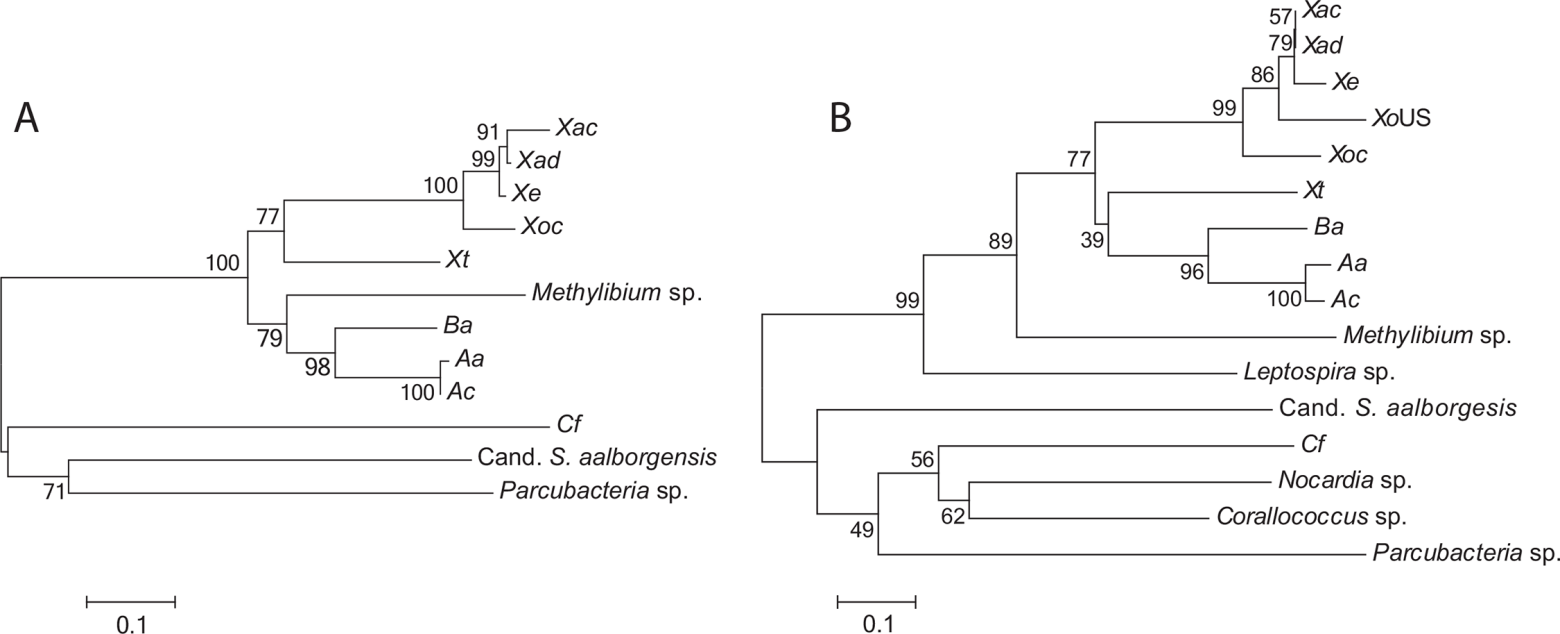
Phylogeny of predicted AvrRxo1-like and Arc1-like proteins in plant pathogenic and environmental bacteria. Neighbor-Joining tree of aligned amino acid sequences from 12 AvrRxo1 homologs (**A**) and 16 Arc1 homologs (**B**). Sequence accessions and strains are listed in Tables 1 and 3. The datasets consisted of 260 (**A**) and 74 (**B**) amino acids after gap elimination. Bootstrap percentages for 1000 replicates are shown next to branches. Units represent the number of amino acid substitutions per site.

BLAST searches using Arc1-*Xoc* also identified predicted proteins from the fruiting myxobacterium *Corallococcus coralloides*, the soil actinomycete *Nocardia rhamnosiphila*, and the human pathogen *Leptospira broomii* as putative Arc1 homologs (Table 3). All predicted Arc1-like proteins from plant pathogens and environmental strains share a short size (most are 81 to 98 amino acids, with *S*. *aalborgensis* at 121 aa), a secondary structure predicted by Jpred to consist of 3 to 4 short helices, and a novel conserved double tyrosine motif: KExFD(x25-27)(Y/L)ExxYY. The motif is not found in the epsilon antitoxin of the AvrRxo1 structural homolog PezT. In the published structure of Arc1-*Xoc* [13], the motif side chains occupy an internal Arc1 interface between the second helix and the coil connecting the first and second helices, which is not close to an AvrRxo1 binding site and might instead be involved in Arc1 folding. Although putative distant homologs from *Leptospira*, *Nocardia*, and *Corallococcus* are all encoded immediately upstream or downstream of larger predicted genes of unknown function, these predicted genes have no detectable similarity to *avrRxo1*. Neighbor-joining phylogenetic analysis of AvrRxo1 and Arc1 homologs shows that plant pathogen homologs form a clade separate from the homologs from environmental strains, with the exception that the *Methylibium* and *Leptospira* proteins fall within the plant pathogen clade (Fig 3). In both AvrRxo1 and Arc1 based trees, the *Burkholderia* and *Acidovorax* sequences group seperately from the *X*. *oryzae* and *X*. *euvesicatoria* homologs, with *X*. *translucens* homologs falling between the two groups (Fig 3).

### The putative distant homolog of AvrRxo1 from the myxobacterium *Cystobacter fuscus* triggers watersoaking and Rxo1-mediated cell collapse in plants

**Fig 4:**
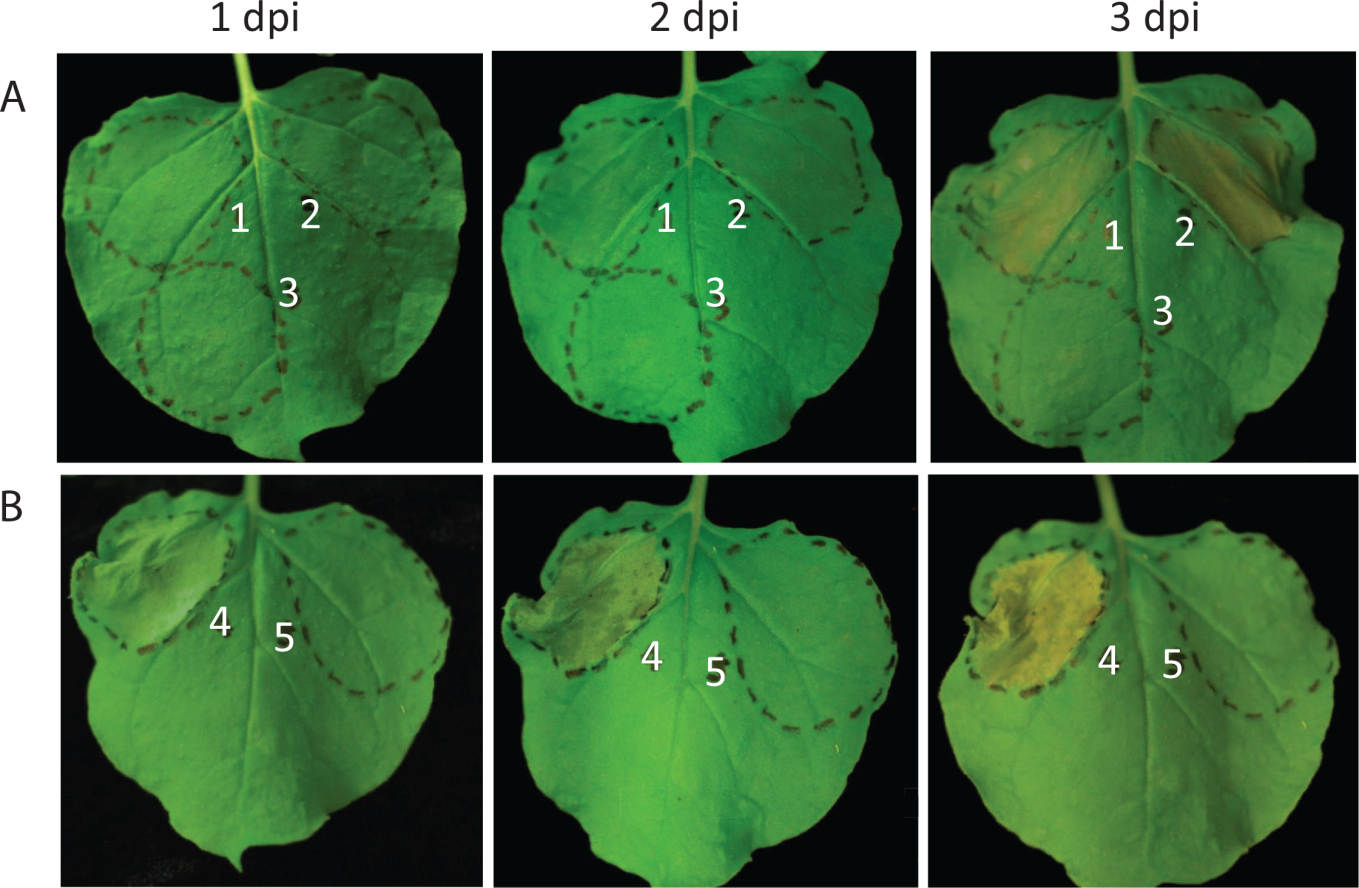
An AvrRxo1 homolog from the myxobacterium *Cystobacter fuscus* causes watersoaking and Rxo1-mediated cell collapse after transient expression in *N*. *benthamiana*. pEG104-AvrRxo1-*Cf* was expressed alone (**A**) and co-expressed with Rxo1 (**B**). 1 = AvrRxo1-Cf (OD600 = 0.1), 2 = AvrRxo1-Cf (OD600 = 0.4), 3 = GFP (OD600 = 0.4), 4 = AvrRxo1-Cf (OD600 = 0.1) and Rxo1 (OD600 = 0.4), 5 = GFP (OD600 = 0.1) and Rxo1 (OD600 = 0.4).

Our above results show that AvrRxo1 homologs from plant pathogens function in bacterial growth suppression, plant toxicity, and Rxo1-mediated hypersensitive response. Given the similarities between these and putative homologs from environmental sequencing projects, we hypothesized that environmental AvrRxo1 homologs might share some or all of these functions. *Cystobacter fuscus* DSMZ2262 was chosen for this study as the only publicly available, non-metagenome-derived environmental strain with a putative *avrRxo1* homolog. We cloned a 1134 nt open reading frame with an alternate GTG start codon, predicted to encode the protein EPX56639, into IPTG and arabinose-inducible expression systems. The gene did not suppress growth of *E*. *coli* under inducing conditions in the arabinose-inducible pBAD expression system (S4 Fig), nor from the IPTG-inducible pDEST527 expression vector. Furthermore, the cloned *arc1-Cf* gene did not relieve the growth suppression of AvrRxo1-*Xoc* (S4 Fig). However, when transiently expressed in *N*. *benthamiana* leaves, a YFP fusion of AvrRxo1-*Cf* caused watersoaking within three days (Fig 4A). When co-expressed with Rxo1, AvrRxo1-*Cf* triggered a rapid cell collapse within one day, consistent with the collapse seen after co-infiltration with AvrRxo1-*Xoc* (Fig 4B). These results demonstrate that, when expressed at high levels *in planta*, a myxobacterial effector homolog is capable of causing a resistance gene-mediated cell collapse.

## Discussion

Once regarded as DNA maintenance modules, TA systems are now thought to increase virulence through mechanisms that may include increased stress resistance, persister cell formation, or biofilm formation [8,23,24]. This study adds two further roles to the repertoire of TA systems, in that AvrRxo1:Arc1 is previously known to function as a type III-secreted virulence factor and as a trigger of effector-triggered immunity. Putative AvrRxo1-like TA systems were also identified in several environmental microbes, and a homolog cloned from the myxobacterium *Cystobacter fuscus* was shown to function as a trigger of the resistance gene Rxo1 in an *N*. *benthamiana* expression system. The identification of *avrRxo1* homologs lacking a predicted T3S signal in environmental bacteria suggests that AvrRxo1 may have an endogenous bacterial role other than virulence.

In the *avrRxo1*:*arc1* module, the antitoxin gene is encoded downstream of the toxin gene, a TA system feature found in relatively few other TA systems [25,26]. The encoding of the antitoxin upstream is thought to ensure that antitoxin expression precedes that of the toxin during translation, preventing unbound toxin from accumulating [27]; upstream toxins thus could potentially pose a selective disadvantage. Here, we find that *avrRxo1* is inactivated in isolates of many species. More study is needed to determine whether *avrRxo1*:*arc1* could be advantageous or disadvantageous to plant pathogenic bacteria outside of the host setting. Intriguingly, in our dataset *avrRxo1* was never inactivated by gene truncation, but always by single site mutagenesis or transposon insertion that left the *arc1* gene intact. Future studies will determine whether *arc1* itself may have a selective advantage.

We observed a diversity of responses to cloned *avrRxo1* homologs in *E*. *coli* and *N*. *benthamiana*. In bacteria, *avrRxo1*-*Xoc* had a relatively weak growth suppression phenotype compared to homologs from *Burkholderia*, *X*. *euvesicatoria* and *X*. *translucens*. No colonies resulted from transformation of the AvrRxo1-*Ac* expression vector into the expression strain after several attempts, leading us to hypothesize that leaky expression of the *Acidovorax* homolog could be particularly toxic to bacteria. Contrary to our expectations, the *avrRxo1* homologs with the greatest bacteriostatic activity did not cause any watersoaking or phytotoxic effects in *N*. *benthamiana* in the absence of Rxo1, but rather only the two homologs with very weak to no bacteriostatic activity (-*Xoc* and–*Cf*) caused watersoaking. It may be that some aspect of strong *avrRxo1* activity diminishes the watersoaking phenotype, or that toxicity in plants and bacteria is caused by two different mechanisms. All *avrRxo1* homologs except *avrRxo1*-*Ac* caused a rapid cell collapse in the presence of Rxo1, suggesting that R-gene triggering activity is broadly conserved in diverse homologs. The basis of these phenotypic differences is not clear from the aligned sequences of AvrRxo1, as there are numerous points of diversity among the homologs (S5 Fig). While the five residues with predicted involvement in ATP binding are completely conserved among the homologs (Fig 1B, S5 Fig), four of the five residues predicted to face the substrate binding site vary among homologs (Fig 1C, S5 Fig). Alternatively, residues outside the active site could influence *in planta* activity by affecting unknown interactions.

Type III-secreted effectors (T3E) have been proposed to originate from the recombinatorial shuffling of domain modules, such as those containing regulatory sequences and secretion signals, onto previously existing genes [28]. However, little is known about the original function of the genes forming the template for new effectors. The structural and functional similarity of AvrRxo1:Arc1 to the zeta:epsilon system suggests that TA toxins could form one source of recombinatorial template for generation of T3E. This hypothesis has already been suggested by the crystal structure of the TA toxin Doc, which bears a strong structural similarity and hypothesized common ancestor with the T3E AvrB [29]. To our knowledge, T3Es have not been systematically tested for their endogenous bacterial function, nor have most TA toxins been tested for secretion activity, so there could be undiscovered functional parallels between the two groups.

Here, we show that an AvrRxo1 homolog cloned from soil myxobacterium can trigger a rapid cell death response in tobacco mediated by a canonical Nucleotide Binding-Leucine Rich Repeat resistance protein. Further study is needed to determine whether a secretion-enabled form of AvrRxo1-CF could trigger resistance in a native infection system. There are few known connections between phytopathogen virulence effectors and environmental bacteria, but advances in whole-genome metagenomics and single-cell sequencing are likely to change that in the near future. For example, studies have recently revealed proteins similar to *Xanthomonas* TAL effectors in unculturable marine organisms [30]. Of the environmental bacteria predicted to encode *avrRxo1* and/or *arc1*-like genes, it is striking that the *Methylibium* strain is a root endophyte, *Endozoicomonas* and *Nocardia* species are marine symbionts, and the greatly reduced genomes of unculturable *Parcubacteria* and *Saccharimonas* spp. have hallmarks of ectosymbionts or ectoparasites [31,32]. It is tempting to think that the presence of *avrRxo1*-like genes in these organisms could be connected with a role in a symbiotic lifestyle.

## Materials and Methods

### Plasmid construction and generation of *E*. *coli* and *Agrobacterium* strains

Primers and plasmid constructs used in this work are listed in S3 Table. *C*. *fuscus* strain DSM2262 was obtained from the DSMZ collection (www.dsmz.de) and cultured on DSMZ medium 222 for three weeks; DNA was then extracted using the Dneasy Blood and Tissue Kit (Qiagen). *avrRxo1* genes from *Xoc* strain BLS256 and *C*. *fuscus* were amplified for cloning into pBAD33 using primers XocBADF and R and CfBADF and R, respectively. PCR products were digested with *Xba*I and *Hin*dIII and cloned into the vector pBAD33, obtained from the Coli Genetic Stock Center (http://cgsc.biology.yale.edu/), to generate pBAD33 (*avrRxo1-Xoc*) and pBAD33 (*avrRxo1-Cf*). Positive clones were confirmed by insert sequencing.

*arc1-Xoc* and *arc1-Cf* were amplified using the primers XocArc1ENTR_F and R, and CfArc1ENTR_F and R. PCR products were cloned into pENTR-D-Topo (Life Technologies) according to the manufacturers’ instructions, and the inserts of confirmed constructs were recombined into pDEST527 (Addgene) using the LR Clonase II enzyme mix (Invitrogen) to generate the plasmids pDEST(*arc1-Xoc*) and pDEST(*arc1-Cf*).

For expression in pDEST527 and comparative growth curves, *avrRxo1* homologs from plant pathogens were amplified from bacterial DNA using primers XeENTR_F and -R (*Xe* strain Xcv85-10), XtENTR_F and -R (*Xt* strain UPB468), BaENTRF and R (*Ba* strain Ba3549), and AcENTR_F and -R (*Ac* strain AAC00-1), and CfENTR_F and R (*Cf* strain DSM2262). PCR products were cloned into pENTR-D-Topo (Life Technologies) and recombined into pDEST527 using the LR Clonase II enzyme mix according to manufacturers’ instructions. pDEST527 constructs were fully sequenced and transformed into *E*. *coli* strain BL21(DE3), and transformants were confirmed by PCR.

### TA system validation assays

Chemically competent *E*. *coli* strain BL21 (DE3) was co-transformed with pBAD33-*avrRxo1(Xoc)* and either pDEST527-*arc1(Xoc)* or pDEST527cv. Transformation was confirmed using colony PCR of both inserts. For bacterial growth curve analysis, transformants were grown in three replicate starter cultures for approximately 6 h at 37°C in M9 medium (KH_2_PO_4_ (22 mM), Na_2_HPO_4_ (90 mM), NH_4_Cl (19 mM), NaCl (9 mM), MgCl_2_ (2mM), CaCl_2_ (0.1 mM)) supplemented with 1% casamino acids, 1% glucose, 100 μg/mL ampicillin and 25 μg/mL chloramphenicol. The bacterial cultures were then adjusted to 10^6^ CFU/mL (OD _600_ = 0.001) in M9 media amended with 1mM IPTG and 1% arabinose or glucose, and each culture was distributed into three wells of a 96-well plate for a total of nine wells per treatment. Cultures were grown in 200 uL volumes at 37°C under continuous orbital shaking in a Synergy H2 microplate reader (Biotek), and OD_600_ was measured every 15 min. The same procedure was used to study pBAD33-*avrRxo1(Cf)*.

### Sequencing and sequence analysis

The genome of *B*. *andropogonis* strain Ba3549 was sequenced in the same run and assembled using the same methods as previously reported for the *US*Xo strain X11-5A [33]. The draft genome was deposited in NCBI under the accession number AYSW00000000.1. The *avrRxo1* gene from Ba3549 was resequenced and deposited in NCBI under accession number KR139796. The *avrRxo1* pseudogene from *X*. *oryzae* X11-5A was amplified using primers USXoF and USXoR, cloned into pCR4-Topo vector (Life Technologies) as per manufacturer’s instructions, and resequenced using the T7 primer to confirm the inactivating mutation.

BLASTP, BLASTn and PsiBLAST searches were conducted in January 2014 and repeated in December 2015. BLASTn was used to query *avrRxo1-Aa* and *avrRxo1-Xo* against the WGS database, limited by group identifiers *Burkholderiaceae*, *Xanthomonadaceae*, *Enterobacteriaceae*, or Myxococcales, using default settings. Psi-BLAST was run for two iterations; only hits with e-value = < 2e-40 and query coverage > 50% were further analyzed. Loci were examined for predicted gene organization using the NCBI genome browser for each draft genome. Sequence alignment and neighbor-joining phylogenetic analysis was conducted in MEGA6 [34] with the default settings.

### *avrRxo1* homolog activity assays

Fresh overnight cultures of BL21(DE3) strains carrying pDEST527-based vectors were suspended at 10^7^ CFU/mL or 10^6^ CFU/mL (OD_600_ of 0.01 or 0.001, respectively) in four replicate tubes of LB broth containing 100 μg/mL ampicillin and 1 mM IPTG. Cultures were distributed into four wells each of a 96 well plate, totaling 16 replicate cultures per treatment. Cultures were incubated at 37°C with shaking, and OD_600_ was measured every two hours for 12 h.

### PCR and Southern hybridization assays for distribution analysis

*Xanthomonas* strains for inoculation or transformation were grown at 28°C on PSA medium. *Burkholderia* and *Acidovorax* strains were grown for DNA extraction at 28° in Nutrient Broth. DNA was extracted from the bacterial strains shown in S1 Table using a standard CTAB extraction method [35]. Conserved primers XoampF and -R and were used to amplify the *avrRxo1* open reading frames from *X*. *oryzae* and *X*. *euvesicatoria* DNA, primers XtENTR_F and–R were used for *X*. *translucens*, and primers BaampF and -R were used to amplify *Acidovorax* and *Burkholderia* DNA. Reactions were performed in a 25 μL solution containing 1x NEB Standard Taq buffer, 200 μM dNTPs, 0.5 μM forward and reverse primer, 20 ng genomic DNA template, and 1 unit Taq polymerase (NEB). After a 30 second initial denaturing step, reactions were cycled 30 times at 98°C for 15s, 57°C for 30s, and 72°C for 90s.

Southern hybridization probes were made using the DIG probe synthesis kit (Roche) according to manufacturer’s instructions, with probe amplification using Ac strain AAC00-1 and Ba strain Ba3549 DNA as templates and primers BaprobeF and–R. 2 μg DNA from selected strains was digested with *Eco*RI or *Hin*dIII as indicated, separated on a 1% agarose gel, and transferred to a nylon membrane (Roche) using a standard capillary transfer protocol [36]. Membranes were hybridized at 42°C for 18 h in 20 ng/mL probe solution in standard buffer, washed four times with standard buffer, and incubated with 1:200 solution of CDP-STAR substrate (Sigma). Membranes were exposed to X-ray film for 1 min before development.

### Plant assays

B73 and Mo17 maize were grown to the five leaf stage in a growth chamber at 26°C. Fully expanded leaves were inoculated with cell suspensions of bacteria (approximately 1 × 10^8^ CFU/ml) prepared from 48- to 72-h-old PSA plate cultures, by infiltration under the leaf with a needleless syringe as previously described [37].

For *in vitro* expression assays, AvrRxo1*-Xoc*, -*Xe*, -*Ba*, -*Ac*, and -*Cf* were recombined from pENTR-D-Topo into pEarleygate 101 or 104 binary vectors for YFP fusion expression (S3 Table). *Agrobacterium tumefaciens* strain GV2260 was transformed with expression plasmids and cultured on LB medium with kanamycin for two days before resuspending in 10 mM MgCl_2_ at an OD_600_ of 0.1 or 0.4. Bacterial solutions were infiltrated into leaves of 6-week old *N*. *benthamiana* plants using a needleless syringe. Plants were incubated in 24 h daylight conditions for at least 48 h before imaging. To study interaction with Rxo1, the single-exon *Rxo1* gene was amplified using the primers Rxo1F and Rxo1R from the genomic DNA of maize line B73, cloned into pENTR-D Topo, and recombined into the pEarleygate 101 vector. Rxo1 expression strains of *Agrobacterium* were infiltrated at OD600 = 0.4.

## Supporting Information

S1 Fig The region downstream of *avrRxo1*-inactivating insertions does not accumulate mutations. Match/mismatch graphs from tBLASTn output of AvrRxo1-*Xoc* sequence queries against subject genomes in which the *avrRxo1* is activated by transposon insertional mutagenesis. **A**) AvrRxo1-*Ac* query against *Acidovorax citrulli* strain tw6 draft genome (sequence ID: JXDJ01000014); **B**) AvrRxo1-*Xoc* query against *Xanthomonas oryzae* pv. *oryzicola* strain CFBP7342/BAI11 complete genome (sequence ID: CP007221).
(EPS)

S2 Fig Insertional mutagenesis and absence of *avrRxo1* gene in some strains revealed by Southern Blot. Southern blots using *avrRxo1* probe against DNA extracted from selected strains of *Acidovorax avenae* (**A**) and *Burkholderia andropogonis* (**B**).
(EPS)

S3 Fig Expression of AvrRxo1 in pEarleygate vectors visualized by confocal microscopy. 1 = pEG104-AvrRxo1-Xoc; 2 = pEG101-AvrRxo1-Ba; 3 = pEG101-AvrRxo1-Ac; 4 = pEG101-AvrRxo1-Xe; 5 = pEG104-AvrRxo1-Cf; and 6 = pEG101 vector.
(EPS)

S4 Fig **AvrRxo1-*Cf* does not cause a decrease in growth of BL21(DE3) cells when expressed from pBAD33 (A), and Arc1-*Cf* does not complement the growth suppressive phenotype of AvrRxo1-*Xoc* (B). A.** Growth of *E*. *coli* BL21(DE3) transformed with pBAD33-*avrRxo1*(*Cf*) or pBAD33 alone under conditions of *avrRxo1* induction (arabinose) or repression (glucose). **B.** Growth of *E*. *coli* BL21(DE3) co-transformed with pBAD33-*avrRxo1* (*Xoc*) and either pDEST-*arc1* (*Cf*) or a pDEST control vector (cv) under the same conditions. Data points and error bars represent the means and standard deviations of 9 independent cultures grown from three starter cultures. Growth experiments were performed three times on separate days with similar results.
(EPS)

S5 Fig Alignment of AvrRxo1 homolog sequences with the secondary structure of AvrRxo1-*Xoc*. The amino acid sequences of AvrRxo1*-Xoc*, *-Xe*, *-Xt*, *-Ba*, *-Ac*, and *-Cf* were aligned using T-Coffee (www.tcoffee.org) and visualized against secondary structural features using ESPript 3.0 (espript.ibcp.fr). Residues contacting ATP are boxed in red, and residues corresponding to the substrate-binding site of zeta toxin are highlighted in yellow.
(PDF)

S1 Table Presence of *avrRxo1* in sequenced bacterial genomes. (DOCX)

S2 Table Responses of B73 (Rxo1+) and Mo17 (Rxo1-) maize to selected bacterial strains, and PCR presence/absence of the *avrRxo1* gene. (DOCX)

S3 Table Primers and plasmid constructs used in this study. (DOCX)
