# Supplementary material for: AvrRxo1 Is a Bifunctional Type III Secreted Effector and Toxin-Antitoxin System Component with Homologs in Diverse Environmental Contexts
Source: PLoS One. 2016 Jul 8;11(7):e0158856. doi: 10.1371/journal.pone.0158856 (PMC4938570; doi:10.1371/journal.pone.0158856)
Supplement: S5 Fig — The amino acid sequences of AvrRxo1-Xoc, -Xe, -Xt, -Ba, -Ac, and -Cf were aligned using T-Coffee (www.tcoffee.org) and visualized against secondary structural features using ESPript 3.0 (espript.ibcp.fr). Residues contacting ATP are boxed in red, and residues corresponding to the substrate-binding site of zeta toxin are highlighted in yellow. (PDF) [file pone.0158856.s005.pdf]

```
Xoc
    1                                                              10        20        30        40        50
Xoc MKNKTDIAG.............................IAGLSTSHVSFDDAESKSVLDGSTQSTPEYKFQTPLDQLPA
Xe  MGVRVAVRIATNVAESVGLYFLGLWTFFNIKNKIGIAGTAGLFTSHVPFDDAESKGVQAESSQAAPEYKFQTQLDNLPA
Xt  MKGKISNACKPDITPI.........................LVDGLASTATQGAADDSASRLPQQALLDGLQE
Ba  MEIQTLYNLQAYDS...................................KSTGSWSEAHTLQDGLPA
Ac  MTDRLSRRNSANSTPR.........................PVTSNAASISRHETPPRPTSRPVHALPAGFPA
Cf  MLAMVRRRSPP..........................PYDDLSKVIANSG..................

                                                      α1            α2      η1
Xoc
          60        70        80        90       100       110       120
Xoc RPAGPKSSNTTDAYIHESLPHSVPIAGASTSAVTHQQATQRKGTDEIYGLGSLPSAGPGRWEYLANPGNWHPERRKLHE
Xe  RPASSKSSNTTHAYVHASPPHSVPIAGASTAAVTHQQATQLKGTDELYGLGNLPSSGPGRWEYLANPENWHPERRKLHE
Xt  RPQALKKSNSAD.WSARTLPQWAPGPGTSSKALLIQSPPHPTSSDEMFGLGELPAPGKARWEYLTKTENWHPERQELHS
Ba  KSQNLVRSSRSQSPVRASLPAGCSAPAMSTSKLAKGLPPETRQSDEIFGLGTLPSFGPSRWDYLAKPENWHPDRRQLHD
Ac  PSQR..SRSSSPSRVRASLPAKVPET..SGNRPAIRLPAAPRGSDEIFGLGELPPPGQSRWDYLAHPQNWRPERRQLHD
Cf  ........................GKLSTTEVFSN.PAEG.......HQGRKPNYAQT.ERFLLQPGNWHPERAQIQQ

       α3                         β1        TT    α4        α5           TT       α6
Xoc
   130       140       150       160       170       180       190       200
Xoc KLLDQARSSALTLAESLESDGCQPTLFALRGNTATGKTRIATKKIPVLAAALKKTAGKGCVNPDDVFKSSL...AKSETG
Xe  KLLDEARSSALTLAESLERDGCQPTLFALRGNTATGKTRIATKKIPALAAALEKTGGKGCINPDDVFKSSL...AKSEAG
Xt  RLLSQARTASLRLAESIESDGYPPTLFALRGNTATGKTRIATKTIPVLANALKKSSGGGCINPDDIFKRSL...AESQGE
Ba  RLIGESKAVAHNFAEAIERGGHPPTLFALRGNTATGKTRMAKQTIPVLAKALKES.DAGCINPDDIFKRAL...AEIPGK
Ac  RLIGDAKTAAQDFAEVIERGGHPPTLFALRGNTAAGKTRMATQTIPVLANALKESGGAGCINPDDIFKRSL...AETPEG
Cf  RLGQQRKDAANRLSDVMAAHGHPNTIVAVMGNTAAGKTT.ALRTLDNFAHLGAH..LDGAINPDPIKADLVQLARKPDG

               α7                     TT      β2       α8            β3        α9
Xoc
      210       220       230       240       250       260       270       280
Xoc AKIFSSAQVHSESSFLADRFEGGLRSQKTGSGAIASIVVDKRLSREYEIDSYIQLAKETGRKVELCDIDAPLENSLVGV
Xe  AKIFSSAQVHNESCFLADHFEDGLRSQRTGSGAIASIVVDKRLARAYEIDSYIELAKETGRKVELCDIDAPLENSLTGV
Xt  AMKLSSAQVHAESCVLADRLENELRSQRTTSGAMASILVDKRLANTHEVDSYVSLAKETGRKVELCDIDAPLERSLMGV
Ba  V.KLSSAQVHAESCVLADRLETELRPLKTASGATASMLIDKRLSGAHEIDAYIKLAEETGRKVELCDIDAPLEQSLMGV
Ac  A.KLTSAQVHAESCILADRLEDELRLQKTASGAAISMLVDKRLAGAHEIDAYIKLAQETGRKIELCDIDAPLEQSLMGV
Cf  QNTISHKQAHQEGNVISQRVEYDMLKTKG....SSLVYDKRFAKRHEFSEMLRTAEQHDKKVQIVDIDSGLTRSAVRV

      TT             α10               α11                    η2   β4       TT   β5
Xoc
     290       300       310                           320       330       340
Xoc LQRKPEGEDPRPPYPVVSSGFVAVRSNRMYVID.................RFIADPSLGNYRLFGTAEDGEKVMVASVI
Xe  LQRKPEGEDPRPPYPVVSSGFVAVRSNRMSVID.................RFIADPSLGNYRLFGTAEDGKKVMVASVM
Xt  LQRSPGGDDPRPPYVAVANGFSTVRSNRLDVID.................RFLSNPTLGSYRLFGTNEKGEKVMVASVA
Ba  LQRKPDGDAPRPPYVAVANGFSAIRGNRLDVID.................KFVSKPNLGSYHLFGTDEGGSKVTVASVV
Ac  LQRKPEGDAPRPPYVAVANGFSAVRGHRLDVID.................KFLSNPTLGSYHLFGTTGNGSKAMVASVV
Cf  LMRPIDSAEPRVPFNAVAEGFIGTRVNREEVLRGRPDEVASDGTRVRGFKGVIDNPRVTSYDLFVPDNKGTPVRVAYKR

      TT β6   α12          η3      α13           α14              α15
Xoc
       350       360       370       380       390       400       410       420
Xoc ....GGEFSVENAELYEKITSPQLSVTGDLADK.VIDKELIDRLENNIADPERAAKTRAALEKYSGKSWSAALAAHSEL
Xe  ....GGELSVEDADLYEKITSPQSSVTGDLADK.IIDNELIDRLTNNIDDPERAANTRAALEKYNGKSWSAALAAHSEL
Xt  ....DGELSIHDPQLYESITAPEDFNSGDKGEQ.VIDTALIDRLSNSIDDPKRATDARAALEKYKGMTWSNALAAHSSL
Ba  ....NGELTIHDPQIYERITDLQGGTAYDVGDQ.VISPELIDRLTKGIDDPARATDARAALERYAGMTWSAALATHSSL
Ac  ....NGELSIHDPQLYEAITDPQGARAGDSGDQ.IIDAALIDRLTKGIDDPARAMDARTALERYAGMTWSDALAAHSSL
Cf  DGVWHGPKTQEQEQLFDRSVKSNPYKSVEVARRIVIDSNFIHKQVEEAPEAFK.APMREALSRFQGMTLEQALDAHSRK

Xoc

Xoc .I
Xe  .I
Xt  .I
Ba  .T
Ac  .T
Cf  IN
```
