# Supplementary material for: AvrRxo1 Is a Bifunctional Type III Secreted Effector and Toxin-Antitoxin System Component with Homologs in Diverse Environmental Contexts
Source: PLoS One. 2016 Jul 8;11(7):e0158856. doi: 10.1371/journal.pone.0158856 (PMC4938570; doi:10.1371/journal.pone.0158856)
Supplement: S1 Table — (DOCX) [file pone.0158856.s006.docx]

**Table S1.** Presence of *avrRxo1* in sequenced bacterial genomes.

| Species | Strain | Host | *avrRxo1* | *arc1* | Upstream gene | Downstream gene |
| --- | --- | --- | --- | --- | --- | --- |
| *Burkholderia andropogonis* | Ba3549 | Sugarcane | + | + | phage integrase | nd |
|  | ICMP2807 | Sorghum (USA) | - | - | N/A | N/A |
| *Acidovorax citrulli* | AAC00-1 | Watermelon | + | + | cyclodehydratase | polyketide biosynth. prot. |
|  | ZJU1106 | Watermelon (China) | i (186)^a^ | + | cyclodehydratase | polyketide biosynth. prot. |
|  | pslbtw65 | Melon | i (186) | + | cyclodehydratase | polyketide biosynth. prot. |
|  | tw6 | Watermelon | i (290) | + | cyclodehydratase | polyketide biosynth. prot. |
| *Acidovorax avenae* | ATCC19860 | Maize (USA) | + | + | cyclodehydratase | polyketide biosynth. prot. |
|  | RS-1 | Rice (China) | + | + | cyclodehydratase | polyketide biosynth. prot. |
|  | T10-61 | Sugarcane (Argentina) | + | + | cyclodehydratase | polyketide biosynth. prot. |
|  | QZ99-2 | Maize (USA) | + | + | cyclodehydratase | polyketide biosynth. prot. |
|  | QZ78-5 | Maize (USA) | + | + | cyclodehydratase | polyketide biosynth. prot. |
|  | QZNCT3 | Turf (USA) | - | - | N/A | N/A |
|  | QZMOR | Turf (USA) | - | - | N/A | N/A |
|  | QZMDB1 | Turf (USA) | - | - | N/A | N/A |
|  | QZQHB1 | Turf (USA) | - | - | N/A | N/A |
|  | QZNCT3 | Turf (USA) | - | - | N/A | N/A |
|  | QZSA2 | Turf (USA) | - | - | N/A | N/A |
|  | QZSH7 | Turf (USA) | - | - | N/A | N/A |
|  | QZSF12 | Turf (USA) | - | - | N/A | N/A |
|  | QZColB1 | Turf (USA) | - | - | N/A | N/A |
| *Xanthomonas oryzae* pv. *oryzicola* | BLS256 | Rice (Philippines) | + | + | transposase | transposase |
|  | CFBP7342/BAI11 | Rice (Burkina Faso) | i (165) | + | transposase | transposase |
|  | BXOR-1 | Rice (India) | + | + | transposase | transposase |
|  | YM15 | Rice (China) | + | + | transposase | transposase |
|  | CFBP2286 | Rice (Malaysia) | + | + | transposase | transposase |
|  | RS105 | Rice (China) | + | + | transposase | transposase |
|  | L8 | Rice (China) | + | + | transposase | transposase |
|  | BLS279 | Rice (Philippines) | + | + | transposase | transposase |
|  | B8-12 | Rice (China) | + | + | transposase | transposase |
|  | MAI10 | Rice (Mali) | - | - | N/A | N/A |
|  | BAI5 | Rice (Burkina Faso) | - | - | N/A | N/A |
| *Xanthomonas oryzae* XoUS | X11-5A | Rice (USA) | i | + | transposase | transposase |
|  | X8-1A | Rice (USA) | i | nd | nd | nd |
| *Xanthomonas translucens* | DAR61454 | Wheat (Australia) | - | - | N/A | N/A |
|  | ART-Xtg29 | Lolium multiflorum | - | - | N/A | N/A |
|  | DSM 18974 | Barley (USA) | + | + | Hypothetical protein | *folB* |
|  | - | Poa trivialis (Switz.) | - | - | N/A | N/A |
|  | LMG 727 | Arrhenatherum elatius (Switzerland) | - | - | N/A | N/A |
|  | B1 | Barley (USA) | + | + | nd | nd |
|  | B2 | Barley (USA) | - | - | N/A | N/A |
|  | - | Phleum pratense (Norway) | - | - | N/A | N/A |
|  | XT123 | Barley (Canada) | - | - | N/A | N/A |
|  | LB5 | Wheat (USA) | - | - | N/A | N/A |
|  | XT-Rocky | Wheat (USA) | - | - | N/A | N/A |
|  | UPB787 | Barley (Paraguay) | - | - | N/A | N/A |
|  | CFBP 2053 | Orchardgrass (Switz.) | - | - | N/A | N/A |
|  | CS2 | Wheat (USA) | - | - | N/A | N/A |
|  | CS22 | Wheat (USA) | - | - | N/A | N/A |
|  | CR31 | Wheat (USA) | - | - | N/A | N/A |
|  | P3 | Wheat (USA) | - | - | N/A | N/A |
|  | CS4 | Wheat (USA) | - | - | N/A | N/A |
|  | LG54 | Wheat (USA) | - | - | N/A | N/A |
|  | LB10 | Wheat (USA) | - | - | N/A | N/A |
|  | XT130 | unknown (Canada) | - | - | N/A | N/A |
|  | XT5770 | unknown (Canada) | - | - | N/A | N/A |
|  | LW16 | Wheat (USA) | - | - | N/A | N/A |
|  | LG48 | Wheat (USA) | - | - | N/A | N/A |
|  | XT8 | Barley (Canada) | - | - | N/A | N/A |
|  | XT5791 | Wheat (Canada) | - | - | N/A | N/A |
|  | XT5523 | Wheat (Canada) | - | - | N/A | N/A |
|  | Xtu 4699 | Wheat (USA) | - | - | N/A | N/A |
|  | CFBP 2541 | Smooth Brome (USA) | - | - | N/A | N/A |
| *Xanthomonas axonopodis* pv. *dieffenbachiae* | LMG 695 | Anthurium (Brazil) | - | - | N/A | N/A |
|  | LMG 25940 | Anthurium (Brazil) | - | - | N/A | N/A |
|  | LMG 12749 | Philodendron (France) | + | + | restriction endonuclease | hypothetical |
|  | LMG 7399 | Dieffenbachia (unknown) | - | - | N/A | N/A |
| *Xanthomonas alfalfae* | F1 | Citrus (USA) | + | + | restriction endonuclease | hypothetical |
|  | CFBP 3836 | Alfalfa (Sudan) | i (252) | + | restriction endonuclease | hypothetical |
|  | LMG 495 | Alfalfa (India) | i (252) | + | restriction endonuclease | hypothetical |
| *Xanthomonas axonopodis* pv. *allii* | CFBP6369 | Onion (Reunion) | i (220) | + | Restriction endonuclease endonuclease | hypothetical |
| *Xanthomonas euvesicatoria* | 85-10 | Pepper (USA) | + | + | restriction endonuclease | hypothetical |
|  | 683 | Pepper (USA) | + | + | restriction endonuclease | hypothetical |
|  | 684 | Pepper (USA) | + | + | restriction endonuclease | hypothetical |
|  | 685 | Pepper (USA) | + | + | restriction endonuclease | hypothetical |
|  | F4-2 | Pepper (USA) | + | + | restriction endonuclease | hypothetical |
|  | H3-2 | Pepper (USA) | + | + | restriction endonuclease | hypothetical |
|  | 689 | Pepper (USA) | + | + | restriction endonuclease | hypothetical |
|  | 695 | Pepper (USA) | + | + | restriction endonuclease | hypothetical |
|  | G4-1 | Pepper (USA) | + | + | restriction endonuclease | hypothetical |
|  | L3-2 | Pepper (USA) | + | + | restriction endonuclease | hypothetical |
|  | 66b | Pepper (Bulgaria) | + | + | restriction endonuclease | hypothetical |
|  | 83M | Pepper (Macedonia) | + | + | restriction endonuclease | hypothetical |
|  | LMG27970 | Pepper (Belgium) | + | + | restriction endonuclease | hypothetical |
|  | 329 | Pepper (USA) | + | + | restriction endonuclease | hypothetical |
|  | 206 | Pepper (USA) | + | + | restriction endonuclease | hypothetical |
|  | 515 | Pepper (USA) | + | + | restriction endonuclease | hypothetical |
|  | 526 | Pepper (USA) | + | + | restriction endonuclease | hypothetical |
|  | 586 | Pepper (USA) | + | + | restriction endonuclease | hypothetical |
|  | 679 | Pepper (USA) | + | + | restriction endonuclease | hypothetical |
|  | 681 | Pepper (USA) | + | + | restriction endonuclease | hypothetical |
|  | 678 | Pepper (USA) | + | + | restriction endonuclease | hypothetical |
|  | 259 | Pepper (USA) | + | + | restriction endonuclease | hypothetical |
|  | 199 | Pepper (USA) | + | + | restriction endonuclease | hypothetical |
|  | 315 | Pepper (USA) | + | + | restriction endonuclease | hypothetical |
|  | 354 | Pepper (USA) | + | + | restriction endonuclease | hypothetical |
|  | 376 | Pepper (USA) | + | + | restriction endonuclease | hypothetical |
|  | 455 | Pepper (USA) | + | + | restriction endonuclease | hypothetical |
|  | 490 | Pepper (USA) | + | + | restriction endonuclease | hypothetical |
|  | 181 | Pepper (USA) | + | + | restriction endonuclease | hypothetical |

^a^ i indicates an inactivation by single site or insertional mutation. The number of amino acids encoded upstream of the inactivation is listed in parentheses.
