# Supplementary material for: AvrRxo1 Is a Bifunctional Type III Secreted Effector and Toxin-Antitoxin System Component with Homologs in Diverse Environmental Contexts
Source: PLoS One. 2016 Jul 8;11(7):e0158856. doi: 10.1371/journal.pone.0158856 (PMC4938570; doi:10.1371/journal.pone.0158856)
Supplement: S2 Table — (DOCX) [file pone.0158856.s007.docx]

**Table S2.** Responses of B73 (Rxo1+) and Mo17 (Rxo1-) maize to selected bacterial strains, and PCR presence/absence of the *avrRxo1* gene.

| Strain | | Source | | Host plant | | | HR | | | | PCR |  |  |  |  |  |  |
| --- | --- | --- | --- | --- | --- | --- | --- | --- | --- | --- | --- | --- | --- | --- | --- | --- | --- |
|  | |  | |  | | | B73 | | Mo17 | |  |  |  |  |  |  |  |
| *X. oryzae pv. oryzicola* | | | | | | |  | | |  | |  |  |  |  |  |  |
| BLS101 | | JE Leach | | *Oryza sativa* | | | HR^a^ | | N | | + |  |  |  |  |  |  |
| BLS179 | | JE Leach | | *O. sativa* | | | HR | | N | | + |  |  |  |  |  |  |
| BLS222 | | JE Leach | | *O. sativa* | | | HR | | N | | + |  |  |  |  |  |  |
| BLS256 | | JE Leach | | *O. sativa* | | | HR | | N | | + |  |  |  |  |  |  |
| BLS295 | | JE Leach | | *O. sativa* | | | HR | | N | | + |  |  |  |  |  |  |
| BLS303 | | JE Leach | | *O. sativa* | | | HR | | N | | + |  |  |  |  |  |  |
| Xoo44 | | JE Leach | | *O. sativa* | | | HR | | N | | + |  |  |  |  |  |  |
| B-911 | | JE Leach | | *O. sativa* | | | HR | | N | | + |  |  |  |  |  |  |
|  | |  | |  | | |  | |  | |  |  |  |  |  |  |  |
| *B. andropogonis* | | | |  | | |  | | | | |  |  |  |  |  |  |
| 3317PA | | LE Claflin | | unknown | | | HR | | P | | NT |  |  |  |  |  |  |
| 3319PA | | A.K Vidaver | | unknown | | | HR | | P | | - |  |  |  |  |  |  |
| 3390PA | | L.E. Claflin | | *Sorghum bicolor* | | | P | | Ws | | - |  |  |  |  |  |  |
| 3394PA | | L.E. Claflin | | *S. bicolor* | | | HR | | P | | + |  |  |  |  |  |  |
| 3395PA | | L.E. Claflin | | *S. bicolor* | | | HR | | P | | - |  |  |  |  |  |  |
| 3397PA | | LE Claflin | | unknown | | | P | | Ws | | NT |  |  |  |  |  |  |
| 3399PA | | L.E. Claflin | | *S. bicolor* | | | P | | Ws | | NT |  |  |  |  |  |  |
| 3342PA | | ATCC 19309 | | *Stizolobium deeringianum* | | | HR | | P | | + |  |  |  |  |  |  |
| 3442PA | | LE Claflin | | unknown | | | HR | | P | | + |  |  |  |  |  |  |
| 3459PA | | CUCPB 1115 | | *S. bicolor* | | | HR | | P | | - |  |  |  |  |  |  |
| 3492PA | | LE Claflin | | unknown | | | HR | | P | | + |  |  |  |  |  |  |
| 3544PA | | LE Claflin | | *S. bicolor* | | | HR | | P | | NT |  |  |  |  |  |  |
| 3549 PA | | LE Claflin | | *Saccharum officinarum* | | | HR | | P | | + |  |  |  |  |  |  |
| 3558PA | | LE Claflin | | *S. bicolor* | | | P | | Ws | | NT |  |  |  |  |  |  |
| 3561PA | | LE Claflin | | *S. bicolor* | | | HR | | P | | - |  |  |  |  |  |  |
| 3562PA | | LE Claflin | | *Sorghum vulgare* | | | HR | | P | | NT |  |  |  |  |  |  |
| 3564PA | | LE Claflin | | *S. bicolor* | | | HR | | P | | NT |  |  |  |  |  |  |
| 3585PA | | N. Tisserat | | *Limonium* sp. | | | HR | | P | | NT |  |  |  |  |  |  |
| B7a | | N. Tisserat | | unknown | | | HR | | P | | - |  |  |  |  |  |  |
|  | |  | |  | | |  | |  | |  |  |  |  |  |  |  |
| *A. citrulli* | |  | |  | | |  | |  | |  |  |  |  |  |  |  |
| 00-1 | | R.D. Gitaitis | | *Citrus medica* | | | N | | N | | + |  |  |  |  |  |  |
| 92-3 | | R.D. Gitaitis | | *Citrullus lanatus* | | | N | N | | | + |  | | | | |  |
| 92-300 | | R.D. Gitaitis | | *C.lanatus* | | | N | N | | | + ^b^ |  |  |  |  |  |  |
| 92-301 | | R.D. Gitaitis | | *C.lanatus* | | | N | | N | | + |  |  |  |  |  |  |
| 92-305 | | R.D. Gitaitis | | *C.lanatus* | | | N | | N | | + |  |  |  |  |  |  |
| 94-36 | | R.D. Gitaitis | | *C.lanatus* | | | N | | N | | + |  |  |  |  |  |  |
| 94-39 | | R.D. Gitaitis | | *C.lanatus* | | | N | | N | | + |  |  |  |  |  |  |
| 94-48 | | R.D. Gitaitis | | *C.lanatus* | | | N | | N | | + |  |  |  |  |  |  |
| 99-5 | | R.D. Gitaitis | | *Cucumis melo* | | | N | | N | | + |  |  |  |  |  |  |
| 200-6 | | R. Walcott | | *C. melo* | | | N | | N | | + |  |  |  |  |  |  |
| 200-18 | | R. Walcott | | *C. melo* | | | N | | N | | + |  |  |  |  |  |  |
| 200-23 | | R. Walcott | | *C.lanatus* | | | N | | N | | +^b^ |  |  |  |  |  |  |
| 200-30 | | R. Walcott | | *C. melo* | | | N | | N | | + |  |  |  |  |  |  |
| 201-12 | | R. Walcott | | *C.lanatus* | | | N | | N | | + |  |  |  |  |  |  |
| 202-66 | | R. Walcott | | *C. melo* | | | N | | N | | + |  |  |  |  |  |  |
| 203-16 | | R. Walcott | | *C. melo* | | | N | | N | | + |  |  |  |  |  |  |
| 203-65 | | R. Walcott | | Unknown | | | N | | N | | + |  |  |  |  |  |  |
| 205-14 | | R. Walcott | | *C.lanatus* | | | N | | N | | + |  |  |  |  |  |  |
| 205-22 | | R. Walcott | | *C.lanatus* | | | N | | N | | + |  |  |  |  |  |  |
| 205-33 | | R. Walcott | | *C.lanatus* | | | N | | N | | + |  |  |  |  |  |  |
| 206-1 | | R. Walcott | | *C.lanatus* | | | N | | N | | + |  |  |  |  |  |  |
| 206-2 | | R. Walcott | | *C.lanatus* | | | N | | N | | + |  |  |  |  |  |  |
| 206-75 | | R. Walcott | | Unknown | | | N | | N | | + |  |  |  |  |  |  |
| 206-79 | | R. Walcott | | Unknown | | | N | | N | | + |  |  |  |  |  |  |
| 206-80 | | R. Walcott | | Unknown | | | N | | N | | + |  |  |  |  |  |  |
| 206-95 | | R. Walcott | | Unknown | | | N | | N | | + |  |  |  |  |  |  |
| 206-101 | | R. Walcott | | Unknown | | | N | | N | | + |  |  |  |  |  |  |
| 206-102 | | R. Walcott | | Unknown | | | N | | N | | + |  |  |  |  |  |  |
| 206-103 | | R. Walcott | | Unknown | | | N | | N | | + |  |  |  |  |  |  |
| Au-2 | | R. Walcott | | *C. melo* | | | N | | N | | + |  |  |  |  |  |  |
| Saticoy B | | R. Walcott | | *C.lanatus* | | | N | | N | | + |  |  |  |  |  |  |
|  | |  | |  | | |  | |  | |  |  |  |  |  |  |  |
| *A. avenae* | |  | |  | | |  | |  | |  |  |  |  |  |  |  |
| 3302PAv | | ICPB PA 134 | | *Zea mays* | | | N | | N | | - |  |  |  |  |  |  |
| 3307PAv | | L.E. Claflin | | *Z. mays* | | | N | | N | | - |  |  |  |  |  |  |
| 3339PR | | ATCC 19307 | | *Saccharium officinalis* | | | N | | N | | + |  |  |  |  |  |  |
| 3378PR | | NCPPB 3029 | | *Z. mays* | | | N | | N | | + |  |  |  |  |  |  |
| 3425PAv | | ATCC 19882 | | *O. sativa* | | | N | | N | | + |  |  |  |  |  |  |
| 3375PR | | PDDCC 3139 | | *S. officinarum* | | | N | | N | | - |  |  |  |  |  |  |
| 3376PR | | NCPPB 3112 | | *Canna indica* | | | N | | N | | - |  |  |  |  |  |  |
| 3379PR | | NCPPB 522 | | *S. officinarum* | | | N | | N | | - |  |  |  |  |  |  |
| 3431PR | | L.E. Claflin | | Millet | | | N | | N | | + |  |  |  |  |  |  |
| 3445PR | | Hayward | | *S. officinarum* | | | N | | N | | + |  |  |  |  |  |  |
|  | |  | |  | | |  | |  | |  |  |  |  |  |  |  |
| *X. axonopodis vesicatoria* | |  | |  | | |  | |  | |  |  |  |  |  |  |  |
| 65-2 | | J. Leach | |  | | | N | | N | | + |  |  |  |  |  |  |
| 81-23 race 2 | R. E. Stall | | | *Capsicum annuum* |  | N | | | N | | + |  | | |  | |  |
| 82-8 race 1 | R. E. Stall | | | *C. annuum* | | | N | | N | | + |  | |  | |  | |
| 90-14 | | R. E. Stall | | *Solanum lycopersicum* | | | N | | N | | + |  |  |  |  |  |  |
| E3 | | R. E. Stall | |  | | | N | | N | | + |  |  |  |  |  |  |
|  | |  | |  | | |  | |  | |  |  |  |  |  |  |  |
| *X. translucens* | |  | |  | | |  | |  | |  |  |  |  |  |  |  |
| B94 | | N. Tisserat | | Turfgrass | | | NT | | NT | | - |  |  |  |  |  |  |
| B95 | | | N. Tisserat | Turfgrass | | | NT | | NT | | - |  | | |  |  |  |
| NCPPB1837 | | | NCPPB | *Phleum pratense* | | | NT | | NT | | - |  | | |  |  |  |
| NCPPB1934 | | NCPPB | | *Bromus inermis* | | | NT | | NT | | - |  |  |  |  |  |  |
| UPB684 | | Rahimian | | *Hordeum vulgare* | | | NT | | NT | | - |  |  |  |  |  |  |
| BLSB3 | | T. Adhikari | | *H. vulgare* | | | NT | | NT | | - |  |  |  |  |  |  |
| NARK-1 | | N. Tisserat | | *Triticum* sp. | | | NT | | NT | | - |  |  |  |  |  |  |
| UPB763 | | Sands | | *H. vulgare* | | | NT | | NT | | + |  |  |  |  |  |  |
| Barl-2 | | N. Tisserat | | *H. vulgare* | | | NT | | NT | | + |  |  |  |  |  |  |
| XT4402 | | N. Tisserat | | Unknown | | | NT | | NT | | - |  |  |  |  |  |  |
|  | |  | |  | | |  | |  | |  |  |  |  |  |  |  |
| *X. campestris*  pv. *gummisudans* | | JE Leach | |  | | | N | | N | |  |  |  |  |  |  |  |
| *X. c.*  pv. *secalis* | | JE Leach | |  | | | Ws | | N | |  |  |  |  |  |  |  |
| *X. c.*  pv. *cerealis* | | JE Leach | |  | | | N | | N | |  |  |  |  |  |  |  |
| *X. c.*  pv. *pelargonii* | | JE Leach | |  | | | N | | N | |  |  |  |  |  |  |  |
| *X. c.*  pv. *holcicola* 86 | | JE Leach | |  | | | N | | N | |  |  |  |  |  |  |  |
| *X. axonopodis* pv*. citri* F1 | | JE Leach | |  | | | N | | N | |  |  |  |  |  |  |  |
| *X. a.* pv. *allii* JV594 | | JE Leach | |  | | | N | | N | |  |  |  |  |  |  |  |
| *X. a.* pv. *allii* 0177 | | JE Leach | |  | | | N | | N | |  |  |  |  |  |  |  |
| *X. fragariae* | | JE Leach | |  | | | Ws | | P | |  |  |  |  |  |  |  |
| *X. c.*  pv. *mangiferaeindicae* | | JE Leach | |  | | | Ws | | N | |  |  |  |  |  |  |  |
| *B. cepacia* (98-2, 99-2) | | JE Leach | |  | | | N | | N | |  |  |  |  |  |  |  |
| *B. gladioli*  92-3 | | JE Leach | |  | | | P | | Ws | |  |  |  |  |  |  |  |
| *B. gladioli* 01-1 | | JE Leach | |  | | | Ws | | N | |  |  |  |  |  |  |  |
| *B. glumae* (291, 3-002) | | JE Leach | |  | | | Ws | | N | |  |  |  |  |  |  |  |
| *B. cocovenenans* | | JE Leach | |  | | | N | | N | |  |  |  |  |  |  |  |
| *Pseudomonas cichorii* | | JE Leach | |  | | | N | | N | |  |  |  |  |  |  |  |
| *P. fluorescens* | | JE Leach | |  | | | N | | N | |  |  |  |  |  |  |  |
| *P. aeruginosa* | | JE Leach | |  | | | N | | N | |  |  |  |  |  |  |  |
| *P. putida* | | JE Leach | |  | | | N | | N | |  |  |  |  |  |  |  |
| *P.s.* pv. *tomato 224* | | JE Leach | |  | | | N | | N | |  |  |  |  |  |  |  |
| *P.s.* pv. *tomato* DC3000 | | JE Leach | |  | | | N | | N | |  |  |  |  |  |  |  |
| *P. s.* pv*. glycinea* race4 | | JE Leach | |  | | | N | | N | |  |  |  |  |  |  |  |
| *P. solanacearum* uw186 | | JE Leach | |  | | | N | | N | |  |  |  |  |  |  |  |
| *Enterobacter cloacae* | | JE Leach | |  | | | N | | N | |  |  |  |  |  |  |  |
| *Erwinia carotovora* | | JE Leach | |  | | | N | | N | |  |  |  |  |  |  |  |
| *E. chrysanthemi* EC16 | | JE Leach | |  | | | N | | N | |  |  |  |  |  |  |  |
| *E. herbicola* | | JE Leach | |  | | | N | | N | |  |  |  |  |  |  |  |
| *Serratia proteamaculans* | | JE Leach | |  | | | N | | N | |  |  |  |  |  |  |  |
| *Klebsiella pneumoniae* | | JE Leach | |  | | | N | | N | |  |  |  |  |  |  |  |
| *Kluyvera cryocrescens* | | JE Leach | |  | | | N | | N | |  |  |  |  |  |  |  |
| *Pantoea ananatis* | | JE Leach | |  | | | N | | N | |  |  |  |  |  |  |  |
| *Pantoea agglomerans* | | JE Leach | |  | | | N | | N | |  |  |  |  |  |  |  |
| *Curtobacterium flaccumfaciens* | | JE Leach | |  | | | N | | N | |  |  |  |  |  |  |  |

^a.^ HR= Hypersensitive response, Ws= Watersoaking, P= Watersoaking and necrosis, N= no visible response.

^b^ denotes strains in which amplicon was larger than expected.
