# Supplementary material for: AvrRxo1 Is a Bifunctional Type III Secreted Effector and Toxin-Antitoxin System Component with Homologs in Diverse Environmental Contexts
Source: PLoS One. 2016 Jul 8;11(7):e0158856. doi: 10.1371/journal.pone.0158856 (PMC4938570; doi:10.1371/journal.pone.0158856)
Supplement: S3 Table — (DOCX) [file pone.0158856.s008.docx]

**Table S3.** Primers and plasmid constructs used in this study.

| **Primers** | | |
| --- | --- | --- |
| Name | Sequence | |
| XocBADF | GATCTCTAGAAGGAGGAGAGTAATGAAAAACAAGACAGACATTGCT | |
| XocBADR | GATCAAGCTTTCAAATTAGCTCGCTGTGAGC | |
| CfBADF | GATCTCTAGAAGGAGGAGAGTAATGCTGGCCATGGTCCGC | |
| CfBADR | GATCAAGCTTTCAGTTGATCTTCCTGGAGTGGC | |
| XocArc1ENTR_F | CACCATGAAAACTTTGACAGG | |
| XocArc1ENTR_R | TCATGACCACGAGAAAAGTGT | |
| CfArc1ENTR_F | CACCATGAGTGCGAGACAAGAAATCCGTGAC | |
| CfArc1ENTR_R | TCAGCCCGCGTCGTGTTC | |
| cvENTR-F | CACCATGAAAAACAAGACAGACATTGCT | |
| cvENTR-R | CAAACCATAGATCTCATCGGTG | |
| XeENTR_F | CACCGGATCCATGGCATCGCCGCCGCATTCGGTG | |
| XeENTR_R | GAGCTCAATTAGCTCGCTGTGAGCAGCT | |
| XtENTR_F | CACCATGAAAGGAAAGATAAGTAATGCATGCAAGCCAGA | |
| XtENTR_R | TCAGATCAATGAGCTGTGTGCCGCTAGAGCATTTG | |
| BaENTR_F | CACCATGATGTCAAGCGGGCGC | |
| BaENTR_R | TTAAGTCAGTGAACTGTGGGTTGCGAGA | |
| AcENTR_F | CACCGGATCCATGACAGACAGACTCAGCCGGCGA | |
| AcENTR_R | GTCGACAGTCAGTGAACTATGGGCGGCCA | |
| CfENTR_F | CACCGTGCTGGCCATGGTCCGC | |
| CfENTR_R | TCAGTTGATCTTCCTGGAGTGCGC | |
| USXoF | GGAGCGCCGTAAGTTACACCAA | |
| USXoR | GCCGACCATGACTTGCCGTTA | |
| XoampF | AGCGCCGTAAGTTGCACGAA | |
| XoampR | AACAATGTCGGTTGGCAGCC | |
| BaampF | AGCGGCGTCAATTGCATGAC | |
| BaampR | TGGCTCGCGCTGGGTCGTCT | |
| BaprobeF | AGCGGCGTCAATTGCATGAC | |
| BaprobeR | TGGCTCGCGCTGGGTCGTCT | |
| Rxo1F | CACCCCCGGGATGGCAGAGATTGCTGTTCTTCT | |
| Rxo1R | GTCGACCATTTCCTTTTGAAAGCTGCTT | |
| **Plasmids** | | |
| Name | Description ^a^ |  |
| pBAD33-*avrRxo1*(*Xoc*) | pBAD33 with the *avrRxo1* ORF from *X. oryzae* pv. *oryzicola* strain BLS256, CmR | |
| pDEST-*arc1* | pDEST527 with the *arc1* ORF from *X. oryzae* pv. *oryzicola* strain BLS256, AmpR | |
| pDESTcv | pDEST527 with a short fragment of the 5' end of *avrRxo1* (reference 12) | |
| pDEST-*avrRxo1*(*Xoc*) | pDEST527 with the *avrRxo1* ORF from *X. oryzae* pv. *oryzicola* strain BLS256, AmpR (reference 12) | |
| pDEST-*avrRxo1*(*Xe*) | pDEST527 with the *avrRxo1* ORF from *X. euvesicatoria* strain 85-11, AmpR | |
| pDEST-*avrRxo1*(*Ba*) | pDEST527 with the *avrRxo1* ORF from *B. andropogonis* strain Ba3549, AmpR | |
| pDEST-*avrRxo1*(*Ac*) | pDEST527 with the *avrRxo1* ORF from *A. citrulli* strain AAC00-1, AmpR | |
| pDEST-*avrRxo1*(*Xt*) | pDEST527 with the *avrRxo1* ORF from *X. translucens* strain UPB468, AmpR | |
| pDEST-*avrRxo1*(*Cf*) | pDEST527 with the *avrRxo1* ORF from *C. fuscus* strain DSMZ2262, AmpR | |
| pEG104-*avrRxo1*(*Xoc*) | pEarleygate 104 binary vector with the *avrRxo1* ORF from *X. oryzae,* KmR (reference 12) | |
| pEG101-*avrRxo*1(*Xe*) | pEarleygate 101 binary vector with the *avrRxo1* ORF from *X. euvesicatoria,* KmR | |
| pEG101-*avrRxo1*(*Ba*) | pEarleygate 101 binary vector with the *avrRxo1* ORF from *B. andropogonis* | |
| pEG101-*avrRxo1*(*Ac*) | pEarleygate 101 binary vector with the *avrRxo1* ORF from *A. citrulli* | |
| pEG104-*avrRxo1*(*Cf*) | pEarleygate 104 binary vector with the *avrRxo1* ORF from *C. fuscus* | |
| pEG101-*Rxo1* | pEarleygate 101 binary vector with the *Rxo1* ORF from maize cDNA | |
| pBAD33-*avrRxo1*(*Cf*) | pBAD33 with the *avrRxo1* ORF from *C. fuscus*, CmR | |
| pDEST-*arc1*(*Cf*) | pDEST527 with the *arc1* ORF from *C. fuscus*, AmpR | |

^a.^ All plasmids are from this study unless a reference is given.
